# Digoxin and Amiodarone on the Risk of Ischemic Stroke in Atrial Fibrillation: An Observational Study

**DOI:** 10.3389/fphar.2018.00448

**Published:** 2018-05-07

**Authors:** Kuan-Cheng Lai, Sy-Jou Chen, Chin-Sheng Lin, Fu-Chi Yang, Cheng-Li Lin, Chin-Wang Hsu, Wen-Chen Huang, Chia-Hung Kao

**Affiliations:** ^1^Department of Emergency Medicine, Tri-Service General Hospital, National Defense Medical Center, Taipei, Taiwan; ^2^Graduate Institute of Injury Prevention and Control, College of Public Health and Nutrition, Taipei Medical University, Taipei, Taiwan; ^3^Division of Cardiology, Department of Internal Medicine, Tri-Service General Hospital, National Defense Medical Center, Taipei, Taiwan; ^4^Department of Neurology, Tri-Service General Hospital, National Defense Medical Center, Taipei, Taiwan; ^5^Management Office for Health Data, China Medical University Hospital, Taichung, Taiwan; ^6^College of Medicine, China Medical University, Taichung, Taiwan; ^7^Department of Emergency Medicine, School of Medicine, Taipei Medical University, Taipei, Taiwan; ^8^Department of Emergency and Critical Medicine, Wan Fang Hospital, Taipei Medical University, Taipei, Taiwan; ^9^Graduate Institute of Clinical Medical Science, School of Medicine, College of Medicine, China Medical University, Taichung, Taiwan; ^10^Department of Nuclear Medicine and PET Center, China Medical University Hospital, Taichung, Taiwan; ^11^Department of Bioinformatics and Medical Engineering, Asia University, Taichung, Taiwan

**Keywords:** amiodarone, digoxin, ischemic stroke, atrial fibrillation, cohort study

## Abstract

**Purpose:** The present study compared the risk of ischemic stroke in atrial fibrillation (AF) patients receiving digoxin and amiodarone.

**Methods:** A retrospective cohort study was conducted using the longitudinal population-based database of Taiwan’s National Health Insurance program. Patients with AF who received amiodarone or digoxin and were considered to have exposed to study drugs consecutively over 180 days during 2000–2010 were enrolled and divided into three groups: those who received amiodarone, digoxin, and amiodarone plus digoxin. All patients were followed from the index date to the occurrence of ischemic stroke, death, withdrawal from the insurance program, or December 31, 2011. Cox proportional hazard regression models were applied to determine the risk of ischemic stroke and associated risk factors.

**Results:** The amiodarone, digoxin, and amiodarone plus digoxin cohorts comprised 797, 1419, and 376 patients, respectively. Overall, the patients who received digoxin (HR = 1.80; 95% CI = 1.41–2.31) or amiodarone plus digoxin (HR = 2.00; 95% CI = 1.49–2.68) had a higher risk of ischemic stroke, compared with those who received amiodarone. This risk was particularly at CHA_2_DS_2_VASc score of 2–5, but disappeared in those who received clopidogrel in the digoxin cohort. The risk of ischemic stroke in the amiodarone plus digoxin cohort did not differ significantly from that in the digoxin cohort (HR = 1.14; 95% CI = 0.90–1.44).

**Conclusion:** Atrial fibrillation patients receiving digoxin are associated with a higher risk of ischemic stroke than are those receiving amiodarone. It is prudent to assess the stroke risk prior to applying treatment strategy for patients with AF.

**Strengths and Limitations of This Study**

- This study is a population-based design with a completeness and accuracy of data, national coverage in both study and control cohorts. All insurance claims were double-checked by medical specialists for peer review.

- Information about serum levels of the drugs, coagulation status, and types of AF were unavailable in this administrative database.

## Introduction

Atrial fibrillation (AF) is a common arrhythmia associated with increased risk of thromboembolic diseases, particularly ischemic stroke (January et al., 2014). In Taiwan, the incidence rates of AF were 1.68 per 1000 person-years for men and 0.76 per 100 person-years for women; the prevalence of AF is 1.4% in men and 0.7% in women (Chien et al., 2010; Chiang et al., 2016). An excessive ventricular rate and an irregular ventricular beat that is associated with AF may both contribute to clinical symptoms and the risk of stroke in patients with AF. Currently, the major treatment strategies for AF are stroke prevention, heart rate control (beta-blockers, calcium-channel blockers, digoxin), and sinus rhythm maintenance (flecainide, amiodarone, electrical ablation, etc.) as indicated in the international and Taiwan guidelines for the management of AF (January et al., 2014; Chiang et al., 2016; Kirchhof et al., 2016).

Several studies had indicated that no significant differences in prognosis between rate control and rhythm control strategies (Van Gelder et al., 2002; Carlsson et al., 2003). A 2008 study of patients with AF and HF showed that rhythm control dose not superior to rate control with regard to cardiovascular mortality, stroke, or worsening HF (Roy et al., 2008). Subsequent echocardiographic analysis also reported no difference in improvement of cardiac functions between these two strategies at 12 months follow-up (Henrard et al., 2013).

Digoxin is among the rate control agents recommended in long-term AF management, particularly in patients with HF. However, a population-based study, including 38,898 digoxin patients, revealed that digoxin use was associated with greater mortality, comparing to other rate-controlling drugs (Chao et al., 2015). A few clinical studies have also raised concerns regarding digoxin safety in the management of AF (Chang et al., 2013; Ouyang et al., 2015). In another population-based study comparing AF patients treated with and without digoxin, each in the absence of an anticoagulant, the digoxin patients exhibited a 1.4-fold increase in the risk of ischemic stroke (Chao et al., 2014). A meta-analysis on digoxin mortality proposed substantial differences in baseline characteristics between patients in digoxin and non-digoxin groups, indicating a higher mortality risk of digoxin in observational studies but a neutral effect in randomized trials and patients on admission for incident stroke (Ziff et al., 2015). Regardless of the contradictive findings on the association of stroke risk with digoxin among these studies, concerns about the potential risk of digoxin remain (Washam et al., 2015).

Nevertheless, amiodarone has also been associated with an increased risk of stroke (Flaker et al., 2014; Chen et al., 2015). Amiodarone is an antiarrhythmic medication that is often applied for rate control and sinus maintenance for patients with AF. A population study comparing patients with AF treated with or without amiodarone suggested that patients who received amiodarone were at 1.8-fold increase in the risk of stroke; notably, digoxin also demonstrated a 1.77-fold increase in the risk of stroke (Chen et al., 2015).

Because of the inconsistent findings regarding stroke risk associations and the similarity of stroke risk results shown in some prior studies on these two commonly used agents for AF management, the objective of the current study was to examine the risk of stroke between AF patients who received amiodarone and digoxin. We hypothesized that the risk for ischemic stroke in the 2 digoxin cohorts is not different from that in the amiodarone cohort.

## Materials and Methods

### Study Design

The study cohort was created using a subset of the NHIRD that contains comprehensive information, including data on inpatients, outpatients, and prescription drugs, from a randomly selected sample of 1 million beneficiaries for the period January 2000–December 2010. Disease diagnosis accorded with the ICD-9-CM. The longitudinal population-based NHIRD comprises claims data for beneficiaries of Taiwan’s National Health Insurance program, which was launched in 1996 and covers more than 99% of the Taiwanese population. The NHIRD is maintained by the National Health Research Institutes [Official website of NHIRD^1^].

### Study Population

We identified newly diagnosed AF patients between January 2000 and December 2010, applying a 6 months wash-up period (July 1999–Dec 1999). Patients with AF (ICD-9-CM code 427.31) who received amiodarone or digoxin over 180 days and were considered to have exposed to study drugs consecutively during January 2000–December 2010 were enrolled in this study and divided into three groups: those who received amiodarone, digoxin, and amiodarone plus digoxin. For each patient, the first date of prescription filling for amiodarone or digoxin was defined as the index date. Patients were excluded if they had ischemic or hemorrhagic stroke (ICD-9-CM codes 430–438), rheumatic heart disease (ICD-9-CM codes 393–398) before the index date or were aged younger than 20 years. Previous ischemic stroke was excluded because it may carry bias in determination of future stroke in the registry databank.

### Baseline Variables

We obtained baseline variables, including age, gender, urbanization levels, occupation, and comorbidities of congestive HF (ICD-9-CM code 428), hypertension (ICD-9-CM codes 401–405), diabetes mellitus (ICD-9-CM code 250), peripheral artery disease (ICD-9-CM codes 440.2, 440.3, 440.8, 440.9, 443, 444.22, 444.8, 447.8, and 447.9), coronary heart disease (CHD, ICD-9-CM codes 410–411), hyperlipidemia (ICD-9-CM code 272), chronic kidney disease (CKD, ICD-9-CM code 585), chronic obstructive pulmonary disease (COPD) (ICD-9-CM codes 491, 492, and 496), and asthma (ICD-9-CM code 493); these comorbidities were considered the potential risk factors of ischemic stroke and were defined before the index date. Furthermore, gastrointestinal bleeding (ICD-9-CM codes 531, 531.4, 532, 532.4, 533, 533.4, 534, 534.4, 535, 535.41, 535.51, 569.3, and 578), medications, (aspirin, clopidogrel, dipyridamole, and warfarin), and medical procedures (cardioversion and transcatheter radiofrequency ablation) along with CHA_2_DS_2_VASc score (congestive HF, hypertension, age ≥ 75, diabetes, previous stroke or transient ischemic attack, vascular disease, age 65 to 74, and female sex) were to adjust the analysis on anticoagulation effect and stroke risk (Lip et al., 2010). HF treatments, including diuretics, β-blockers, calcium channel blockers (CCBs) [dihydropyridine (DHP) and non-DHP CCBs], angiotensin converting enzymes inhibitors, and angiotensin II receptor blockers were applied to adjust the analysis on HF severity. The NHIRD stratified all city districts and townships in Taiwan into 7 urbanization levels, based on population density (people/km^2^), proportion of residents with higher education, elderly and agricultural population, and the number of physicians per 100,000 people in each area. Level 1 represented areas with a higher population density and socioeconomic status, and level 7 represented the lowest. Because few people lived in more rural areas of levels 4–7, our study grouped these areas into the level 4 group (Liu and Liu, 2010). The occupation categories included public servants, workers in the labor sector (farmers, fishermen, and industry workers), businessmen, low-income earners, and others. A low income was defined as a monthly income lower than the level required for paying a premium.

### Outcome Measurement

All patients were followed-up from the index date to the occurrence of ischemic stroke (ICD-9-CM codes 433–438) or until the patients were censored because of death, withdrawal from the insurance program, or December 31, 2011.

### Statistical Analysis

Distribution of age, sex, comorbidities, and medications were compared among the three groups. A chi square test was conducted to evaluate the differences in baseline characteristics, except for mean age, which was examined using one-way ANOVA. Cox proportional hazards regression models was used to investigate the risk of stroke among groups. In the model, with the time duration from baseline assessment to the occurrence of the first stroke as the dependent variable, HRs with a 95% CI were calculated. The gender, age, urbanization level, occupation, CHA_2_DS_2_VASc score, comorbidities, medications, and medical procedures were adjusted in the Cox models. The risks of stroke among groups were stratified by CHA_2_DS_2_VASc score and antithrombotic medications. We estimated group-specific cumulative incidences by using Kaplan–Meier survival curves, with significance based on the log-rank test. All data analyses were performed using SAS Version 9.3 (SAS Institute Inc., Cary, NC, United States). *P* < 0.05 was considered statistically significant.

### Data Availability Statement

The dataset used in this study is held by the Taiwan Ministry of Health and Welfare (MOHW). The Ministry of Health and Welfare must approve our application to access this data. All relevant data are within the paper. Any researcher interested in accessing this dataset can submit an application form to the Ministry of Health and Welfare requesting access. Please contact the staff of MOHW for further assistance.^2^

### Ethics Statement

The NHIRD encrypts patient personal information to protect privacy and provides researchers with anonymous identification numbers associated with relevant claims information, including sex, date of birth, medical services received, and prescriptions. Therefore, patient consent is not required to access the NHIRD. This study was approved to fulfill the condition for exemption by the Institutional Review Board (IRB) of China Medical University (CMUH104-REC2-115-CR2). The IRB also specifically waived the consent requirement.

## Results

Comparison of the baseline characteristics of the patients with AF who received amiodarone, digoxin, and amiodarone plus digoxin was shown in **Table 1**. The patients who received digoxin were older on average than those in the other two groups. Approximately 35% of the patients who received digoxin lived in the least urbanized region among all patients, whereas 28% of those who received amiodarone lived in the least urbanized region. Among the cohorts, the highest proportions of patients with hyperlipidemia and CKD were in the amiodarone cohort; moreover, the highest proportions of β-blocker consumption as well as reception of transcatheter radiofrequency ablation was associated with this cohort. The highest proportions of patients with congestive HF, COPD, asthma, diuretics consumption as well as high CHA_2_DS_2_VASc score (≥4) were in the digoxin cohort; the highest proportion of patients with CHD, warfarin consumption, and reception of cardioversion were in the amiodarone plus digoxin cohort.

**Table 1:** Comparison of demographics and comorbidity among atrial fibrillation (AF) patients treated with amiodarone or digoxin.

	Atrial fibrillation						*p*-value
	Amiodarone (*N* = 797)		Digoxin (*N* = 1419)		Amiodarone and digoxin (*N* = 376)		
	*n*	(%)	*n*	(%)	*n*	(%)	
**Age, year**							<0.001
≤64	268	(33.6)	327	(23.0)	130	(34.6)	
65–74	236	(29.6)	411	(29.0)	127	(33.8)	
≥75	293	(36.8)	681	(48.0)	119	(31.7)	
Mean (*SD*)^∗^	69.2 (12.4)		72.6 (12.1)		68.7 (11.1)		<0.001
**Sex**							0.45
Female	316	(39.7)	600	(42.3)	159	(42.3)	
Male	481	(60.4)	819	(57.7)	217	(57.7)	
**Urbanization level^†^**							0.01
1 (highest)	217	(27.2)	334	(23.5)	88	(23.4)	
2	219	(27.5)	335	(23.6)	108	(28.7)	
3	137	(17.2)	257	(18.1)	56	(14.9)	
4 (lowest)	224	(28.1)	493	(34.7)	124	(33.0)	
**Occupation**							0.003
Public	110	(13.8)	152	(10.7)	50	(13.3)	
Labor	331	(41.5)	625	(44.1)	167	(44.4)	
Business	213	(26.7)	324	(22.8)	95	(25.3)	
Low income	3	(0.38)	21	(1.48)	0	(0.00)	
Others	140	(17.6)	297	(20.9)	64	(17.0)	
**Congestive heart failure**	260	(32.6)	861	(60.7)	211	(56.1)	<0.001
**Hypertension**	658	(82.6)	1110	(78.2)	313	(83.2)	0.01
**Diabetes mellitus**	175	(22.0)	274	(19.3)	83	(22.1)	0.24
**Peripheral artery disease**	64	(8.03)	88	(6.20)	19	(5.05)	0.11
**Coronary heart disease**	594	(74.5)	977	(68.9)	295	(78.5)	<0.001
**Hyperlipidemia**	353	(44.3)	365	(25.7)	111	(29.5)	<0.001
**Chronic kidney disease**	74	(9.28)	74	(5.21)	13	(3.46)	<0.001
**Chronic obstructive pulmonary disease**	256	(32.1)	590	(41.6)	142	(37.8)	<0.001
**Asthma**	123	(15.4)	312	(22.0)	78	(20.7)	<0.001
**GI bleeding**	492	(61.7)	819	(57.7)	228	(60.6)	0.16
**CHA_2_DS_2_VASc score**							<0.001
0–1	92	(11.5)	91	(6.41)	26	(6.91)	
2–3	255	(32.0)	404	(28.5)	128	(34.0)	
4–5	359	(45.0)	697	(49.1)	172	(45.7)	
>5	91	(11.4)	227	(16.0)	50	(13.3)	
**Antithrombotic**							
Aspirin	710	(89.1)	1187	(83.7)	329	(87.5)	0.001
Clopidogrel	240	(30.1)	305	(21.5)	113	(30.1)	<0.001
Dipyridamole	494	(62.0)	873	(61.5)	230	(61.2)	0.96
Warfarin	147	(18.4)	295	(20.8)	94	(25.0)	0.03
**Diuretics**	469	(58.9)	1116	(78.7)	267	(71.0)	<0.001
**β-Blockers**	683	(85.7)	1016	(71.6)	313	(83.2)	<0.001
**CCB (non-DHP or DHP)**	705	(88.5)	1210	(85.3)	330	(87.8)	0.08
**ACEI/AIIRB**	631	(79.2)	1172	(82.6)	307	(81.7)	0.14
**Cardioversion**	33	(4.14)	17	(1.20)	17	(4.52)	<0.001
**Transcatheter radiofrequency ablation**	13	(1.63)	4	(0.28)	5	(1.33)	0.002

*^∗^One way ANOVA test. ^†^The urbanization level was categorized by the population density of the residential area into four levels. CHA2DS2VASc score = congestive heart failure, hypertension, age 75 years or older (doubled risk weight), diabetes, previous stroke or transient ischemic attack (doubled risk weight), vascular disease, age 65 to 74, and female sex.*

**FIGURE 1:**
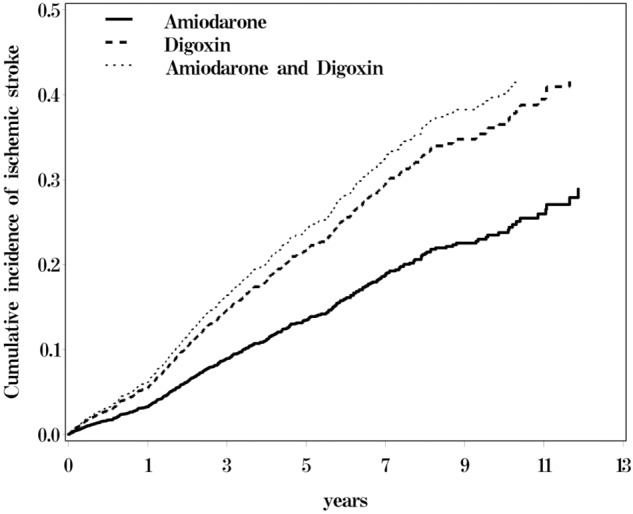
Cumulative incidence of ischemic stroke for atrial fibrillation (AF) patients receiving amiodraone, digoxin, or amiodraone plus digoxin.

**Figure 1** shows the cumulative incidence of ischemic stroke among the three cohorts of AF patients, with the patients who received only digoxin and those who received both amiodarone and digoxin exhibited a higher risk of ischemic stroke than did those who received only amiodarone.

The incidence rate of ischemic stroke per 100 person per year was 2.65, 5.09, and 5.21 for the amiodarone, digoxin, and amiodarone plus digoxin cohorts, respectively, with a mean follow-up of 4.45, 3.86, and 4.95 years (**Table 2**). A higher risk of ischemic stroke was shown in AF patients who received digoxin (HR = 1.80; 95% CI = 1.41–2.31) or amiodarone plus digoxin (HR = 2.00; 95% CI = 1.49–2.68), compared with those who received amiodarone alone. After stratification, AF patients who received digoxin or amiodarone plus digoxin with respective CHA_2_DS_2_VASc score of 2–3 and 4–5 were associated with a higher risk of ischemic stroke, compared with those who received only amiodarone. The stroke risk remained greater in AF patients concomitantly using antithrombotic medications or anticoagulant in the digoxin and amiodarone plus digoxin cohorts. However, the risk was disappeared in those who received clopidogrel in the digoxin cohort.

**Table 2:** Incidence and Cox model-measured hazard ratio (HR) of ischemic stroke by CHA_2_DS_2_VASc score and medications in study subjects.

	Amiodarone (*N* = 797)		Digoxin (*N* = 1419)		Amiodarone and digoxin (*N* = 376)		Adjusted HR^§^ (95% CI)	
	Event	Rate^#^	Event	Rate^#^	Event	Rate^#^	Digoxin vs. amiodarone	Amiodarone and digoxin vs. amiodarone
**All**	94	2.65	279	5.09	97	5.21	1.80 (1.41, 2.31)^∗∗∗^	2.00 (1.49, 2.68)^∗∗∗^
**CHA_2_DS_2_VASc score**								
0–1	5	1.01	10	2.12	2	1.30	2.17 (0.54, 8.78)	0.97 (0.14, 6.55)
2–3	21	1.57	61	3.25	34	4.78	2.29 (1.37, 3.86)^∗∗^	3.55 (2.02, 6.24)^∗∗∗^
4–5	53	3.65	155	6.25	50	6.21	1.49 (1.07, 2.07)^∗^	1.69 (1.13, 2.52)^∗^
> 5	15	5.60	53	8.10	11	5.78	1.48 (0.80, 2.72)	0.92 (0.40, 2.10)
**Antithrombotic**								
Aspirin	95	2.76	269	5.18	93	4.78	1.77 (1.37, 2.29)^∗∗∗^	1.84 (1.34, 2.51)^∗∗∗^
Clopidogrel	30	2.66	39	2.69	27	4.13	1.04 (0.60, 1.79)	1.88 (1.06, 3.35)^∗^
Dipyridamole	70	2.94	219	5.87	77	5.83	1.89 (1.42, 2.53)^∗∗∗^	2.12 (1.50, 3.02)^∗∗∗^
Warfarin	18	2.07	72	4.22	35	5.24	2.15 (1.14, 4.04)^∗^	2.97 (1.48, 5.97)^∗∗^

*^∗^p < 0.05, ^∗∗^p < 0.01, ^∗∗∗^p < 0.001. ^#^Rate: incidence rate, per 100 person-years. ^§^Adjusted HR: multivariable analysis including urbanization level, occupation, comorbidity of hyperlipidemia, chronic kidney disease, chronic obstructive pulmonary disease, asthma, and GI bleeding, medication of diuretics, β-blockers, CCB, ACEI, AIIRB, cardioversion, and transcatheter radiofrequency ablation.*

Furthermore, the risk of ischemic stroke was compared between the patients who received digoxin and those who received both amiodarone and digoxin (**Table 3**). Overall, the difference in the risk of ischemic stroke was insignificant between these patients. However, patients who received both drugs had a higher risk of ischemic stroke than those who only received digoxin at CHA_2_DS_2_VASc score 2–3 (HR = 1.56, 95% CI = 1.00–2.43).

**Table 3:** Hazard ratio of ischemic stroke for AF patients receiving both amiodarone and digoxin, applying the digoxin cohort as a reference.

	Amiodarone and digoxin (*N* = 376)	
	Unadjusted HR	Adjusted HR^§^
	(95% CI)	(95% CI)
**All**	1.05 (0.83, 1.32)	1.14 (0.90, 1.44)
**CHA_2_DS_2_VASc score**		
0–1	0.70 (0.15, 3.18)	1.03 (0.15, 7.00)
2–3	1.47 (0.97, 2.24)	1.56 (1.00, 2.43)^∗^
4–5	1.04 (0.76, 1.43)	1.14 (0.83, 1.59)
>5	0.69 (0.36, 1.33)	0.52 (0.25, 1.08)

*^∗^p < 0.05. ^§^Adjusted HR: multivariable analysis including urbanization level, occupation, comorbidity of hyperlipidemia, chronic kidney disease, chronic obstructive pulmonary disease, asthma, and GI bleeding, medication of aspirin/ clopidogrel, dipyridamole, warfarin, diuretics, β-blockers, CCB, ACEI, AIIRB, cardioversion, and transcatheter radiofrequency ablation.*

The interaction of clopidogrel and CHA_2_DS_2_VASc score on the risk of ischemic stroke was shown in **Table 4**. The risk of ischemic stroke was insignificant in patients who received clopidogrel in the amiodarone and the digoxin cohorts, compared with those who had no clopidogrel at CHA_2_DS_2_VASc score of 0–1 in the amiodarone cohort. However, increase in the risk of stroke was shown for those who received clopidogrel in the amiodarone plus digoxin cohort at CHA_2_DS_2_VASc score of 2–3 (HR = 3.81; 95% CI = 1.16–12.5), and 4–5 (HR = 3.25; 95% CI = 1.04–10.2).

**Table 4:** Cox proportional hazard regression analysis of the risk of ischemic stroke with the interaction of CHA_2_DS_2_VASc score and clopidogrel.

Medication	CHA_2_DS_2_VASc score	Rate^#^	Adjusted HR^§^
			(95% CI)
**Without clopidogrel**			
Amiodarone	0–1	1.00	Reference
	2–3	1.50	1.23 (0.40, 3.74)
	4–5	4.08	2.99 (1.05, 8.54)^∗^
	>5	6.26	4.27 (1.30, 14.0)^∗^
Digoxin	0–1	2.40	2.05 (0.64, 6.59)
	2–3	3.49	2.69 (0.96, 7.52)
	4–5	7.58	5.25 (1.90, 14.5)^∗∗^
	>5	10.2	6.65 (2.33, 19.0)^∗∗∗^
Amiodarone and digoxin	0–1	1.44	1.36 (0.25, 7.48)
	2–3	4.91	3.86 (1.33, 11.2)^∗^
	4–5	7.18	5.08 (1.77, 14.6)^∗∗^
	>5	6.95	4.79 (1.44, 15.9)^∗^
**With clopidogrel**			
Amiodarone	0–1	1.07	1.29 (0.14,11.6)
	2–3	1.78	1.44 (0.40, 5.16)
	4–5	2.83	1.97 (0.63, 6.11)
	>5	4.63	3.27 (0.85, 12.5)
Digoxin	0–1	0.00	–
	2–3	2.26	1.85 (0.55, 6.21)
	4–5	2.57	1.77 (0.58, 5.38)
	>5	3.77	2.42 (0.71, 8.30)
Amiodarone and digoxin	0–1	0.00	–
	2–3	4.46	3.81 (1.16, 12.5)^∗^
	4–5	4.48	3.25 (1.04, 10.2)^∗^
	>5	3.29	2.56 (0.46, 14.3)

*^∗^p < 0.05, ^∗∗^p < 0.01, ^∗∗∗^p < 0.001. ^#^Rate: incidence rate, per 100 person-years. ^§^Adjusted HR: multivariable analysis including gender, age, urbanization level, occupation, comorbidity of hyperlipidemia, chronic kidney disease, chronic obstructive pulmonary disease, asthma, and GI bleeding, medication of aspirin, dipyridamole, warfarin, diuretics, β-blockers, CCB, ACEI, AIIRB, cardioversion, and transcatheter radiofrequency ablation.*

## Discussion

In this population-based cohort study, we compared the risk of ischemic stroke in patients with AF who received amiodarone, digoxin, and digoxin plus amiodarone. Overall, the ischemic stroke rate was 1.8- and 2.0-fold increase in patients who received digoxin and amiodarone plus digoxin, respectively, comparing with those who received amiodarone. The risks of ischemic stroke for each the digoxin and amiodarone plus digoxin cohorts was significantly higher at CHA_2_DS_2_VASc score of 2–3 and 4–5 compared with the amiodarone cohort. No significant risk difference was observed between AF patients who received digoxin alone and those who received amiodarone plus digoxin.

The association of stroke risk with digoxin has been reported by Chao et al. (2014) in uncoagulated AF patients who did not have HF. Applying amiodarone cohort as control in the present study, a higher risk of stroke remained significant, particularly at CHA_2_DS_2_VASc score of 2–3. These results seem to imply that digoxin is associated with a greater risk of stroke in patients who do not have HF or who are classified as medium stroke risk. However, it should be noted that despite the adjustment for medications associated with HF, the difference in HF severity between the digoxin and amiodarone cohorts may not have been fully accounted for without incorporating an analysis of heart ejection fraction. Nevertheless, a higher risk of stroke consistently present in the digoxin cohorts, suggesting that the indication for digoxin use should balance the potential risk it brings about, particularly for those who are scored at 2–3 or without HF.

Compared with patients who received digoxin alone, patients who received both amiodarone and digoxin did not have a significant greater risk of stroke than those who received digoxin alone, suggesting amiodarone might not add an increased risk for those who are on digoxin treatment. In the effective anticoagulation with factor Xa next generation in the AF–Thrombolysis in Myocardial Infarction 48 (ENGAGE AF-TIMI 48) trial, in which amiodarone was used in 11.8% of patients, no significant risk difference was observed in patients who received amiodarone (Steffel et al., 2015). However, in the Apixaban for Reduction in Stroke and Other Thromboembolic Events in AF trial, in which patients received either warfarin or apixaban, those who received amiodarone were associated with a 1.47-fold increase in the risk of stroke or systemic embolism (Flaker et al., 2014). Moreover, in the population study by Chen et al. (2015), in which 20.2% of the sampled patients underwent anticoagulation therapy, amiodarone was associated with an overall 1.8-fold increase in the risk of stroke. These two studies support that amiodarone increases the risk of stroke; however, the authors acknowledged that patients who received amiodarone are associated with less effective anticoagulation or low anticoagulation rates. Collectively, these findings suggest that amiodarone might not significantly increase the risk of stroke and that the increase in the overall amiodarone-induced stroke risk is associated with the conditions of less effective or low-coverage anticoagulation therapy.

Although the mechanism may not be elucidated by the registry data, the risk of stroke significantly reduced in the patients who received clopidogrel in the digoxin cohort, suggesting that digoxin might play a role in platelet activation (Chirinos et al., 2005). Of note, more than 90% of the patients who received clopidogrel were also on aspirin. However, anti-platelet in patients with AF has limited protective effect against stroke in clinical practices. The lower event rates of stroke may also reflect the benefit of combination antiplatelet therapy or improvement in other factors associated with stroke, such as atherosclerosis, coronary heart disease, and diabetes mellitus.

This study had several limitations. First, data on the serum level concentrations of digoxin and INR were not available to determine the effect of digoxin and warfarin; therefore, we could not assess the possible adverse effects associated with high therapeutic serum digoxin levels and the suboptimal anticoagulation of warfarin (Ahmed et al., 2008). We did not consider non-vitamin K antagonist oral anticoagulants in the study analysis because they were not available in Taiwan during the study period. However, the bleeding risk, including intracranial hemorrhage and GI bleeding, were controlled in the baseline analysis to minimize the anticoagulation discrepancy. Second, the AF type may be different between digoxin and amiodarone users. There may have been more permanent AF patients in the digoxin groups and more paroxysmal AF patients in the amiodarone group. Moreover, despite using amiodarone cohort as the comparison group, adjustment for CHA_2_DS_2_VAS_C_ scores and medical therapy regarding AF, the confounding by indication for AF medications may not be fully eliminated, even by propensity score matching analysis, which was limited in the present study because of a relatively small sample size. Third, we could not assess the daily life activity, serial echocardiographic, or other function assessments from the databank. Patients using digoxin are likely to be frail with compromised cardiopulmonary function, limiting their activity and increasing their stroke risk.

## Conclusion

Atrial fibrillation patients receiving digoxin are associated with a higher risk of ischemic stroke than are those receiving amiodarone. The observational study reveals the necessity of adequate anticoagulation for the management of patients with AF in Taiwan population. It is prudent to assess the stroke risk prior to applying treatment strategy for patients with AF.

## Author Contributions

K-CL and C-HK: conceptualization, investigation, and resources. K-CL, S-JC, C-SL, F-CY, C-LL, C-WH, W-CH, and C-HK: data curation, formal analysis, validation, visualization, writing (original draft preparation), and writing (review and editing). C-HK: funding acquisition, project administration, and supervision. C-LL and C-HK: methodology and software.

## Conflict of Interest Statement

The authors declare that the research was conducted in the absence of any commercial or financial relationships that could be construed as a potential conflict of interest.

## Footnotes

1 http://nhird.nhri.org.tw/en/index.html

2 Mail Address: Taiwan Ministry of Health and Welfare. No. 488, Sec. 6, Zhongxiao E. Rd., Nangang Dist., Taipei City 115, Taiwan (R.O.C.). Phone: +886-2-8590-6848. Email: stcarolwu@mohw.gov.tw.

## Abbreviations

AF: atrial fibrillation
CI: confidence interval
HF: heart failure
HR: hazard ratio
ICD-9-CM: International Classification of Diseases, Ninth Revision, Clinical Modification
NHIRD: National Health Insurance Research Database
